# Diagnostic accuracy of MUAC for assessment of acute malnutrition among children aged 6–59 months in Africa: systematic review and meta-analysis

**DOI:** 10.3389/fnut.2025.1536386

**Published:** 2025-03-10

**Authors:** Yonatan Menber, Tefera Belachew, Netsanet Fentahun

**Affiliations:** ^1^Department of Nutrition and Dietetics, School of Public Health, College of Medicine and Health Sciences, Bahir Dar University, Bahir Dar, Ethiopia; ^2^Department of Nutrition and Dietetics, Faculty of Public Health, Institute of Health, Jimma University, Jimma, Ethiopia

**Keywords:** acute malnutrition, MUAC, WHZ, validation, diagnostic accuracy, children, Africa

## Abstract

**Background:**

Mid-Upper Arm Circumference (MUAC) or Weight-for-Length Z-Score (WHZ) are used to screen for acute malnutrition in children. The relative merits of MUAC and WHZ, as well as whether they ought to be used separately, are still up for debate. Considering the significant impact of acute malnutrition on a large number of children in Africa, along with the constraints on resources, it is crucial to critically assess the validity of simple and widely used tools utilized in both African communities and clinical settings. Therefore, this study aimed to assess the diagnostic test accuracy of MUAC in screening acute malnutrition among children aged 6–59 months in Africa.

**Methods:**

A systematic review and meta-analysis study was conducted to pool evidence on the diagnostic performance of MUAC compared to WHZ among children aged 6 to 59 months across various studies in Africa. The StataMP 17.0 software was utilized for analysis, employing a Bivariate Random-effects Meta-Analysis model. Sensitivity, specificity, the Diagnostic Odds Ratio, and the Area Under the Curve were calculated. Heterogeneity was assessed using Cochrane’s Q statistic and the I^2^ test. Additionally, meta-regression, subgroup analysis, sensitivity analysis, and assessments for publication bias were employed. The overall level of diagnostic test accuracy was estimated using a random-effects meta-analysis model.

**Results:**

Seventeen studies were included in the meta-analysis. The pooled sensitivity and specificity were 38.1% (95% CI: 30.7, 46.1%) and 94.9% (95% CI: 93.2, 96.2%), respectively. The summary receiver operating characteristic curve plot showed that MUAC had good accuracy in detecting acute malnutrition (AUC = 0.85, 95% CI: 0.82, 0.88). The pooled level of diagnostic odds ratio was 13.22 (95% CI: 9.68, 16.77). The rate of misclassification in screening for acute malnutrition using MUAC was observed to be 11.7%.

**Conclusion:**

The MUAC demonstrated low sensitivity but high specificity in diagnosing acute malnutrition in children aged 6 to 59 months across various regions of Africa. Furthermore, it was found that MUAC provides good diagnostic test accuracy when compared to WHZ. To enhance its accuracy, it is suggested to increase the MUAC cutoff thresholds.

## Background

Malnutrition in children is a global issue, particularly in Africa, but often goes underdiagnosed due to diagnostic concerns (1, 2). A low Weight-for-Length/Height z-score (WHZ) or bilateral pitting edema or low Mid-Upper Arm Circumstance (MUAC) are indicators of acute malnutrition (wasting), a type of undernutrition. This falls into two categories: moderate acute malnutrition (MAM) and severe acute malnutrition (SAM), collectively known as Global Acute Malnutrition (GAM). MAM is defined as a MUAC measurement ≥11.5 cm and < 12.5 cm, or a WHZ ≥ −3 SD and < −2 SD, based on World Health Organization (WHO) criteria, for children aged 6–59 months. SAM is diagnosed by bilateral pitting edema or severe wasting (MUAC <11.5 cm or WHZ < −3 SD) (3, 4).

MUAC examinations are a simpler and more affordable alternative for both community and clinical settings than WHZ. WHZ involves weight and height measurements and has barriers in terms of resource availability, portability, and technical abilities, particularly in community setting evaluations. MUAC is less prone to measurement errors and can be completed by a single person. For many years, MUAC assessments have been used to identify acute malnutrition. The WHO recommends utilizing WHZ or MUAC, or a combination of the two, as well as looking for signs of edema, to identify children suffering from acute malnutrition for treatment (3, 5).

The relative merits of MUAC and WHZ, as well as whether they ought to be used separately, are still up for debate. Both WHZ and MUAC identify child groups that overlap but are not identical (2, 6, 7). The poor sensitivity of MUAC at standard cutoffs has serious consequences for program efficacy. Suggesting that the metric is only able to identify just a small proportion of the overall number of wasted children. In the process of treating acute malnutrition, this will also have an impact on case identification, limit the number of children who are eligible for treatment, and ultimately have an impact on the overall rates of admission and discharge. Studies proposed varied ideal MUAC cutoff values for different age groups and sexes rather than having a standard set of cutoff values for all children in the age range of 6–59 months (3, 8).

In nutrition services, proving the agreement between wasting assessment indicators and putting solutions into practice is essential. This might lessen the likelihood that children will be treated inappropriately. Children from diverse ethnic groups with varying body frames require more research, as evidenced by these discrepancies in studies when compared to WHO recommendations (8).

Given that a large number of children in Africa are affected by acute malnutrition, and there are limited resources available, special attention should be paid to the validity of simple and widely used tools in African communities and clinical settings (9). The available evidence of MUAC’s performance versus the WHZ in children varies across different research conducted in Africa (10–13). Summarizing the current evidence in Africa is critical for evidence-based practice in nutrition and health. Therefore, this systematic review and meta-analysis aimed to summarize the currently available evidence on the diagnostic accuracy of MUAC in screening acute malnutrition among children aged 6–59 months in Africa.

## Methods

### Protocol registration and reporting

This review protocol was prepared following Preferred Reporting Items for Systematic Review and Meta-analysis of Diagnostic Test Accuracy Studies (PRISMA-DTA) guidelines.

### Eligibility criteria

The diagnostic accuracy of MUAC (index test) was validated against WHZ (reference standard) for detecting acute malnutrition (the condition of interest) in children aged 6 to 59 months in Africa (target population). This review included diagnostic test accuracy studies published in English that either reported or allowed for the calculation of true positive (TP), false positive (FP), false negative (FN), and true negative (TN) values based on the provided metrics. All cross-sectional studies conducted at the community level, including survey-based research, were considered. Moreover, studies performed at the hospital level that screened all patients, not just those already screened and admitted for malnutrition treatment, were also included. Only studies that utilized the WHO 2006 child growth standards to calculate WHZ were considered.

### Search and study selection

A systematic search was conducted across PubMed/MEDLINE, EMBASE, Scopus, Cochrane Library, and Web of Science databases. In addition, a comprehensive search was undertaken using various sources such as Google Scholar, university repositories, and reference lists of established articles. The search was carried out from its commencement till October 20, 2024.

The search terms used were (“validation” OR “accuracy” OR “performance” OR “predictive ability” OR “diagnostic ability” OR “diagnostic accuracy” OR “precision” OR “reliability” OR “correlation” OR “agreement” OR “sensitivity” OR “specificity”) AND (“MUAC” OR “mid upper arm circumference” OR “mid-upper arm circumference”) AND (“wasting” OR “acute malnutrition” OR “undernutrition” OR “malnutrition”) AND (“infant” OR “child” OR “preschool”) AND (“Algeria” OR “Angola” OR “Benin” OR “Botswana” OR “Burkina Faso” OR “Burundi” OR “Cabo Verde” OR “Cameroon” OR “Central African Republic” OR “Chad” OR “Comoros” OR “Congo” OR “Cote d Ivoire” OR “Democratic Republic Of The Congo” OR “Djibouti” OR “Egypt” OR “Equatorial Guinea” OR “Eritrea” OR “Eswatini” OR “Ethiopia” OR “Gabon” OR “Gambia” OR “Ghana” OR “Guinea” OR “Guinea-Bissau” OR “Kenya” OR “Lesotho” OR “Liberia” OR “Libya” OR “Madagascar” OR “Malawi” OR “Mali” OR “Mauritania” OR “Mauritius” OR “Morocco” OR “Mozambique” OR “Namibia” OR “Niger” OR “Nigeria” OR “Rwanda” OR “Sao Tome and Principe” OR “Senegal” OR “Seychelles” OR “Sierra Leone” OR “Somalia” OR “South Africa” OR “South Sudan” OR “Sudan” OR “Tanzania” OR “Togo” OR “Tunisia” OR “Uganda” OR “Zambia” OR “Zimbabwe”).

### Data collection process

Two independent reviewers evaluated the articles for overall study quality and inclusion in the review. Any ambiguous information or disagreements among the reviewers were handled through consensus. The data extraction tool includes author names, publication year, study area, participant ages, study design, sample size, index test, and reference standard. In addition, the tool contains information on TP, FP, FN, and TN results of MUAC as compared to WHZ.

### Definitions for data extraction

Acute malnutrition was operationally defined with MUAC <12.5 cm as the index test and WHZ < −2 SD as the reference standard. TP was classified as a diagnosis of acute malnutrition based on MUAC, which was confirmed by WHZ. FP was labeled as a diagnosis of acute malnutrition based on MUAC, but it was confirmed by WHZ to be not acutely malnourished. FN was categorized as a diagnosis of not acutely malnourished based on MUAC, which was later confirmed by WHZ to be acutely malnourished. TN was classified as a diagnosis of not acutely malnourished based on MUAC and confirmed by WHZ.

### Risk of bias and applicability

The Quality Assessment of Diagnostic Accuracy Studies-2 (QUADAS-2) tool was used to evaluate study bias and applicability across four domains: patient selection, index test, reference standard, and participant flow and timing using Review Manager 5.3 Software (14). The results were then presented in graphs. The publication bias was checked by Egger’s test and funnel plot of asymmetry using the Diagnostic Odds Ratio (DOR) as effect size.

### Diagnostic accuracy measures and meta-analysis

The extracted data were imported to StataMP 17.0 for further processing and analysis. The *metadta* statistical package was used for meta-analysis (15, 16). Another statistical procedure, *midas*, was employed to calculate the Area Under the Curve (AUC) (16, 17). Sensitivity, specificity, and the Diagnostic Odds Ratio (DOR) were calculated for each study using data from two-by-two tables. The meta-analysis provided pooled estimates of sensitivity, specificity, and DOR, along with 95% confidence intervals, which were visualized through forest plots. A Summary Receiver Operating Characteristic (SROC) plot was constructed using the Bivariate Random-Effects Meta-Analysis (BRMA) model, incorporating 95% confidence and prediction regions. The Area Under the Curve (AUC) was interpreted according to predefined criteria: fail (0.5–0.6), poor (0.6–0.7), fair (0.7–0.8), good (0.8–0.9), and excellent accuracy (0.9–1.0) (18).

The heterogeneity across studies was checked using the Cochrane Q test and Inconsistency index (I^2^) statistic. Additionally, the heterogeneity was also assessed by visual inspection of the paired forest plots and SROC plots. The heterogeneity was declared low, moderate, or high when I^2^ statistic results became 25, 50%, or 75%, respectively. Since all the included studies used the same thresholds for MUAC and WHZ to diagnose acute malnutrition, the use of different thresholds was not a concern for this study, which addresses one of the major sources of heterogeneity in diagnostic accuracy within systematic reviews and meta-analyses. To further investigate heterogeneity, meta-regression and a subgroup analysis were performed considering the available covariates. A sensitivity analysis was conducted to demonstrate the influence of individual studies on the overall estimates of the meta-analysis.

## Results

### Study selection

**Figure 1:**
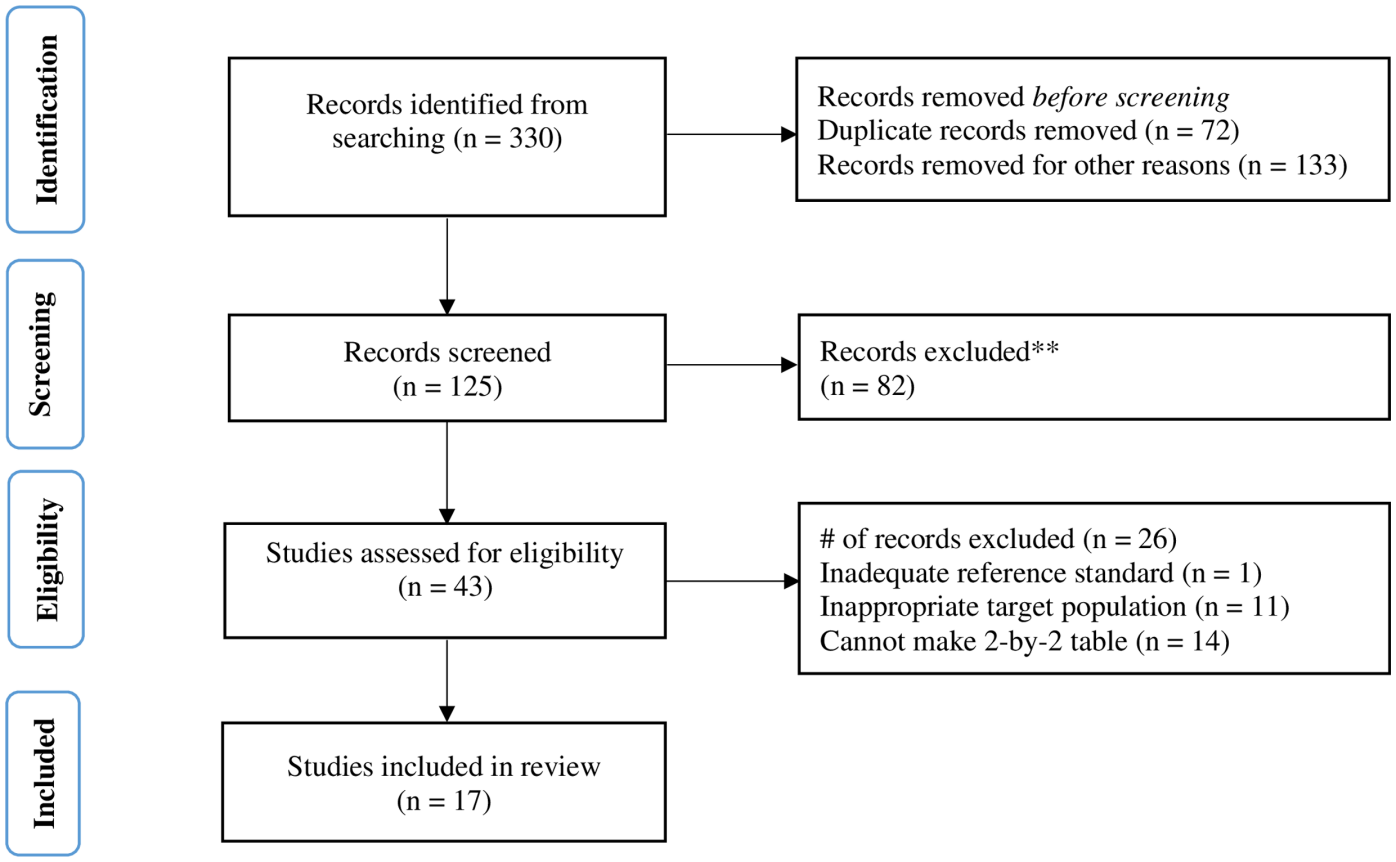
Flow diagram of studies included in the systematic review and meta-analysis.

The search strategy yielded a total of 330 results from published studies. After excluding studies for various reasons, such as duplication, publication dates prior to the development of the WHO 2006 growth standards, and other criteria, 125 studies were retrieved for screening. Of the 43 studies assessed for eligibility, 26 were excluded for various reasons, including issues related to the appropriateness of the reference standard and index test thresholds, the suitability of the target population, and the ability to create a 2-by-2 table. True positive (TP), false positive (FP), false negative (FN), and true negative (TN) results were obtained either directly from the studies or calculated from other provided parameters, such as prevalence, sensitivity, or specificity. Ultimately, 17 studies were included in the meta-analysis (Figure 1).

### Study characteristics

A total of 2,247,021 children were included in this meta-analysis. The minimum sample size was 389 participants in a study conducted in Uganda (11) and the largest sample size was 1,221,352 in a survey conducted in 35 African countries (2). From the total of the reviewed studies, three studies were conducted in Ethiopia (19–21) and the other are conducted in various countries (1, 2, 7, 11, 13, 19–30) (Table 1).

**Table 1:** Summary characteristics of studies included in the meta-analysis.

Article	Study	Population	Country	Setting	Study design	Sample size	Index test (cm)	Reference standard (SD)	TP	FP	FN	TN
1	Ahn (22)	Children aged 0–59 months	South Sudan	Community-based	Cross-sectional	3,358	MUAC <12.5	WHZ < −2	214	220	376	2,548
2	Barro (23)	Children aged 6–59 months	Mauritania	Community-based	Cross-sectional	12,590	MUAC <12.5	WHZ < −2	364	265	1,660	10,301
3	Calistus (13)	Children aged 0–59 months	Kenya	Community-based	Cross-sectional	1,347	MUAC <12.5	WHZ < −2	103	76	197	971
4	Custodio et al. (24)	Children aged 6–59 months	Somalia	Community-based	Cross-sectional	255,623	MUAC <12.5	WHZ < −2	9,343	10,595	31,697	203,988
5	Custodio et al. (1)	Children aged 6–59 months	26 African countries	Community-based	Cross-sectional	647,197	MUAC <12.5	WHZ < −2	19,007	13,287	47,187	567,716
6	Grellety et al.(2)	Children aged 6–59 months	35 African countries	Community-based	Cross-sectional	1,221,352	MUAC <12.5	WHZ < −2	56,419	54,808	90,076	1,020,049
7	John et al. (25)	Children aged 6–59 months	Nigeria	Facility based	Cross-sectional	413	MUAC <12.5	WHZ < −2	12	16	34	351
8	Lambebo et al. (19)	Children aged 6–59 months	Ethiopia	Community-based	Cross-sectional	914	MUAC <12.5	WHZ < -2	37	18	143	716
9	Marshall et al.(26)	Children aged 6–23 months	Niger	Community-based	Cross-sectional	1,161	MUAC <12.5	WHZ < −2	215	146	56	744
10	Ngaboyeka et al. (27)	Children aged 6–59 months	DRC	Facility based	Cross-sectional	6,960	MUAC <12.5	WHZ < −2	1,121	731	644	4,464
11	Odei et al. 2020 (28)	Children aged 6–59 months	Uganda	Community-based	Cross-sectional	32,962	MUAC <12.5	WHZ < −2	1,544	1945	2,422	27,051
12	Roberfroid et al. (7)	Children aged 6–59 months	Chad and South Sudan	Community-based	Cross-sectional	10,210	MUAC <12.5	WHZ < −2	767	455	1,375	7,613
13	Sendaula et al. (11)	Children aged 6–59 months	Uganda	Facility based	Cross-sectional	389	MUAC <12.5	WHZ < −2	91	7	101	190
14	Tadesse et al. (20)	Children aged 6–59 months	Ethiopia	Community-based	Cross-sectional	4,297	MUAC <12.5	WHZ < −2	132	318	100	3,747
15	Tessema et al. (21)	Children aged 6–59 months	Ethiopia	Community-based	Cross-sectional	25,755	MUAC <12.5	WHZ < −2	738	1,402	1,697	21,918
16	Zaba et al. 2020 (29)	Children aged 6–59 months	Mozambique	Community-based	Cross-sectional	12,639	MUAC <12.5	WHZ < −2	160	308	227	11,944
17	Zaba et al. (30)	Children aged 6–59 months	Mozambique	Community-based	Cross-sectional	9,854	MUAC <12.5	WHZ < −2	115	232	166	9,341

MUAC, mid-upper arm circumference; WHZ, weight-for-length/height Z-score; cm, centimeter; SD, standard deviation; TP, true positive; FP, false positive; FN, false negative; TN, true negative; DRC, democratic republic of congo.

### Risk of bias and applicability

**Figure 2:**
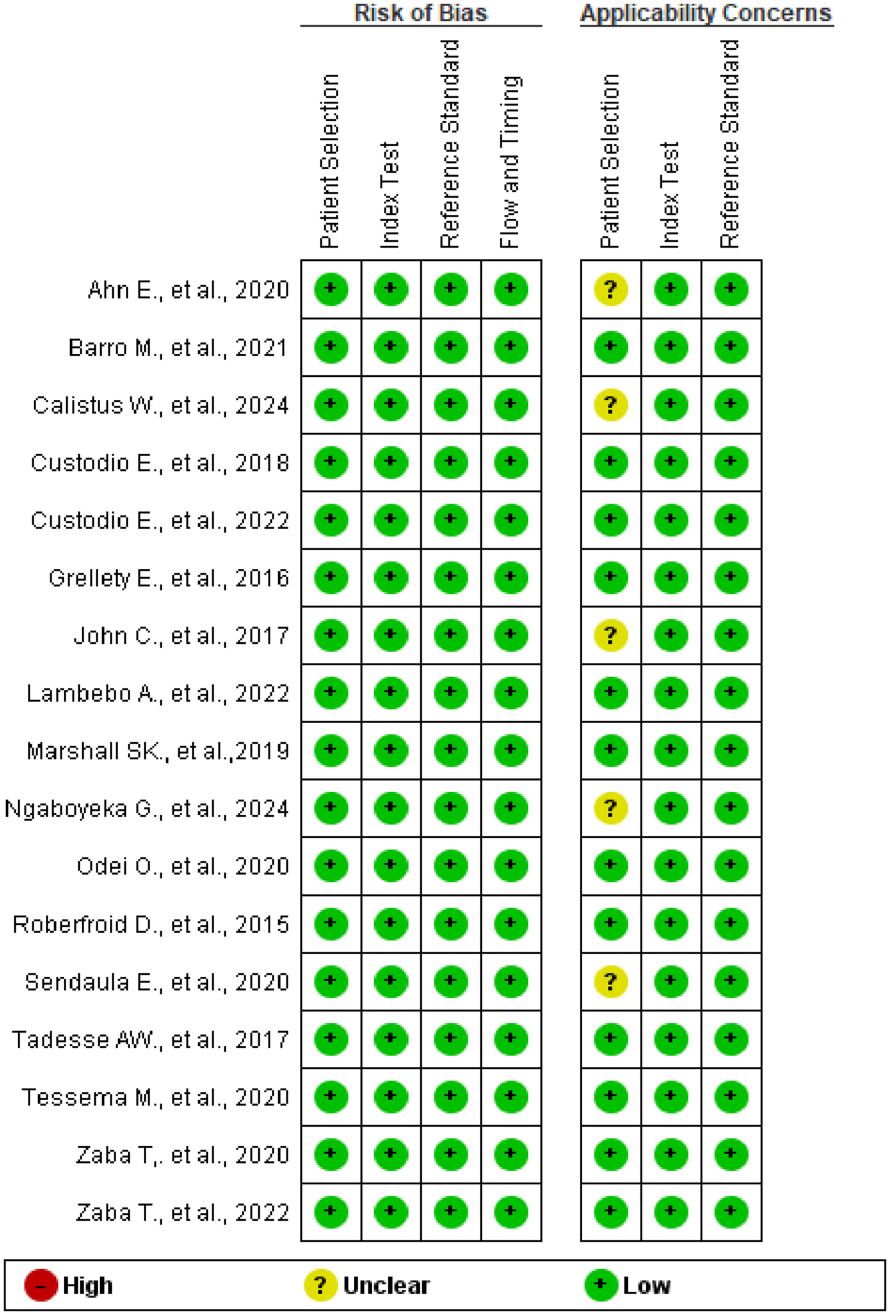
Risk-of-bias and applicability concerns summary.

**Figure 3:**
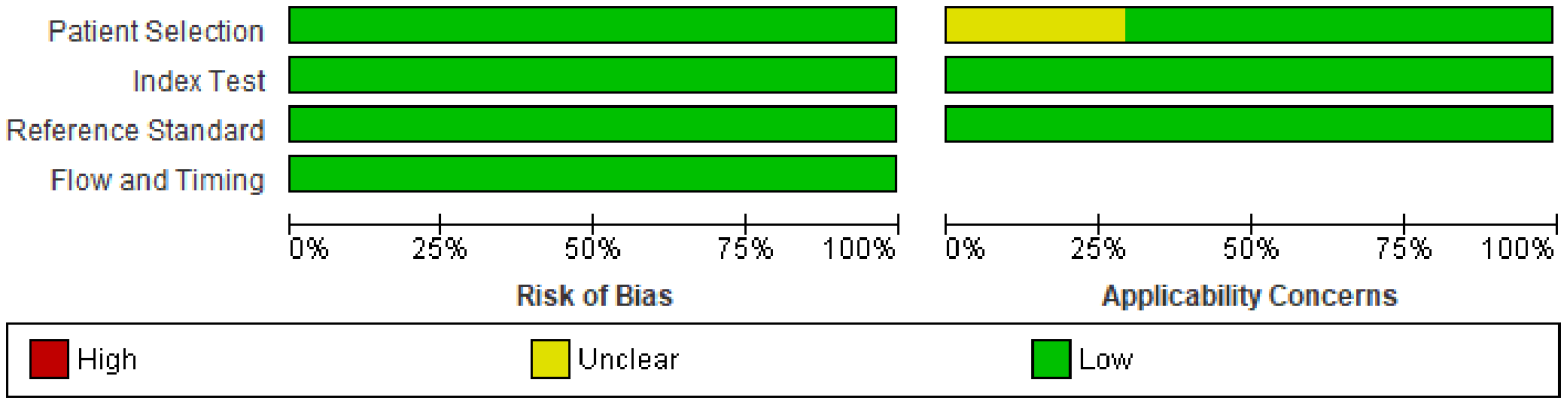
Risk-of-bias and applicability concerns.

According to the QUADAS-2 tool assessment, no risk of bias was detected in any of the studies across the four domains. Regarding applicability concerns, five studies have unclear issues related to participant selection. The concern arises from facility-based studies and the lack of clear information about the inclusion of children under six months of age alongside those aged 6–59 months, for whom inclusion is confirmed (Figures 2, 3).

### Diagnostic accuracy of MUAC

**Figure 4:**
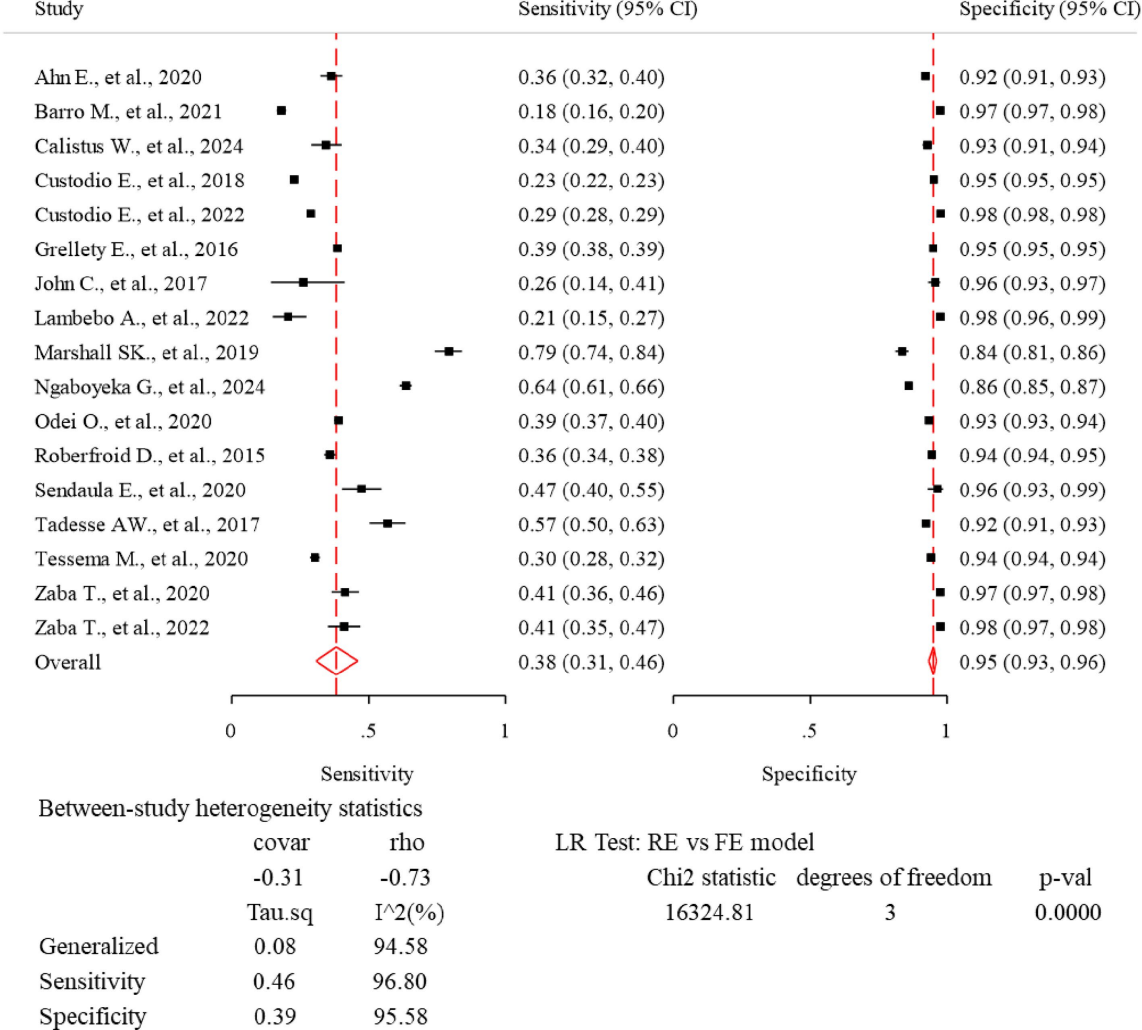
Forest plot—meta-analysis of diagnostic accuracy of MUAC to diagnose acute malnutrition among children aged 6–59 months in Africa.

This review findings revealed that high heterogeneity was detected in the fixed model and the random-effects model provided a better fit to the data (*p*-value <0.0001). The correlation (rho) between sensitivity and specificity on the logit scale was −0.73. There was a nearly equivalent level of heterogeneity in sensitivity (*τ*^2^ = 0.46, *I*^2^ = 96.8%) and specificity (*τ*^2^ = 0.39, *I*^2^ = 95.6%). The generalized between-study heterogeneity without chance as indicated by bivariate *I*^2^ statistic was also high (*τ*^2^ = 0.08, *I*^2^ = 94.58%). There were non-overlapping confidence intervals in the forest plot showing heterogeneity in the magnitude of effects (Figure 4).

**Figure 5:**
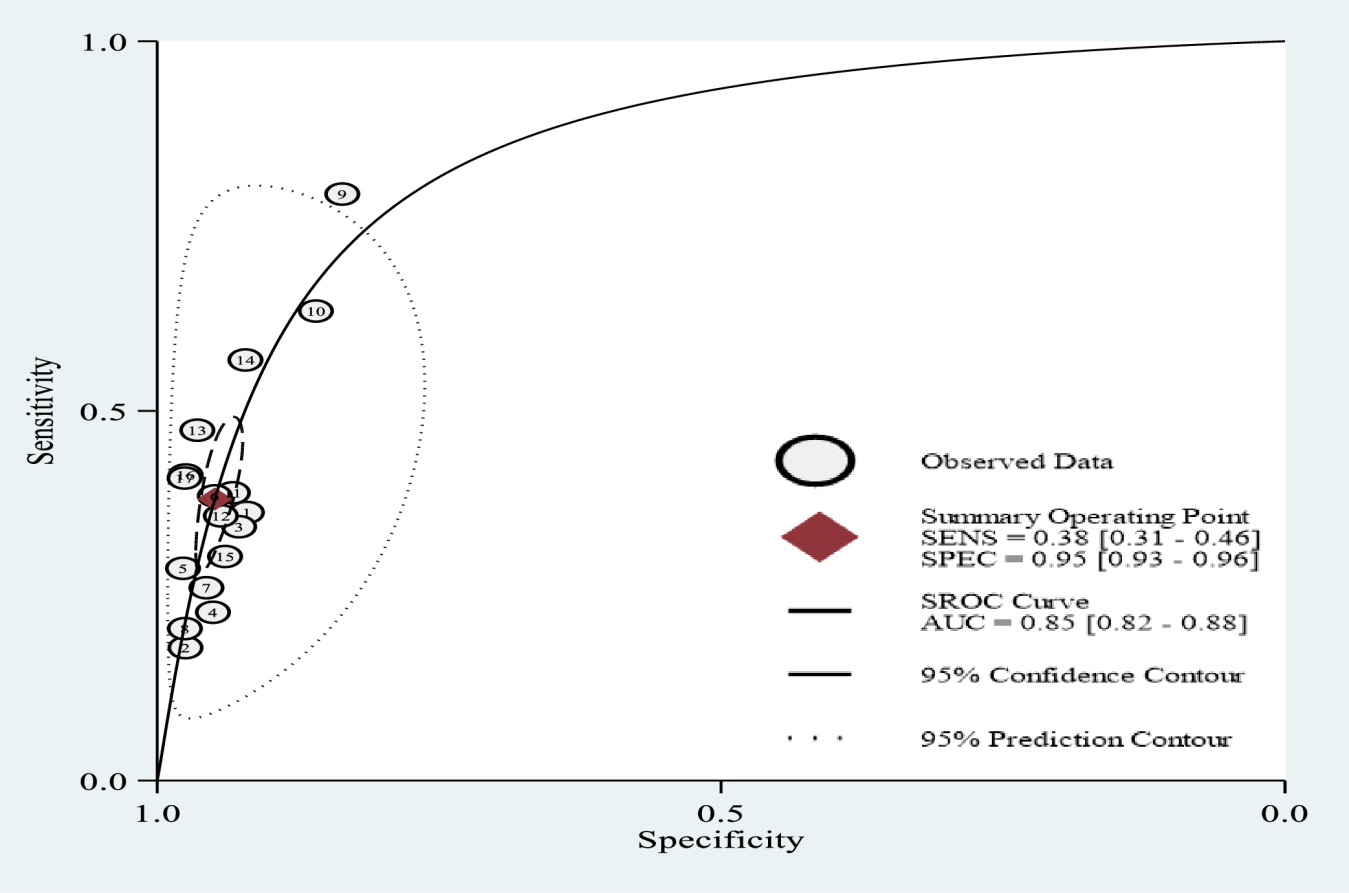
SROC plots from the *midas* statistical package.

**Figure 6:**
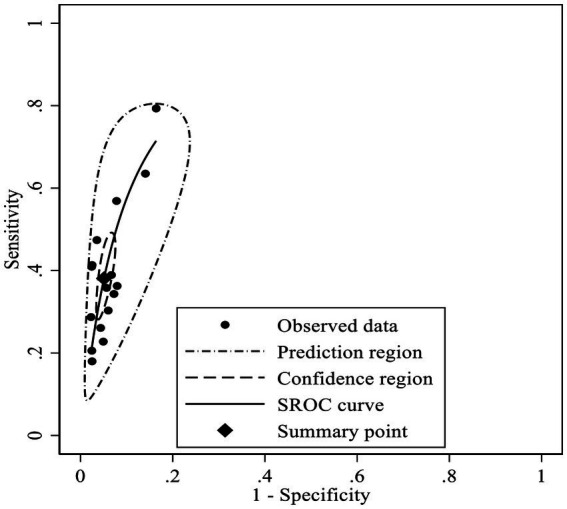
SROC plots from the *metadta* statistical package.

The lowest sensitivity of MUAC for detecting acute malnutrition was 18.0% in Mauritania (23), while the highest sensitivity was 79.3% in Niger (26). The lowest specificity of MUAC to detect acute malnutrition was 83.6% in Niger (26), and the highest specificity was 97.7% in a study conducted in 26 African countries (1). The pooled sensitivity and specificity were 38.1% (95% CI: 30.7, 46.1%) and 94.9% (95% CI: 93.2, 96.2%), respectively (Figure 4). The rate of misclassification in screening for acute malnutrition using MUAC was observed to be 11.7%. The SROC plot showed that MUAC had good accuracy in detecting acute malnutrition (AUC = 0.85, 95% CI 0.82, 0.88) (Figures 5, 6).

### Results based on DOR

**Figure 7:**
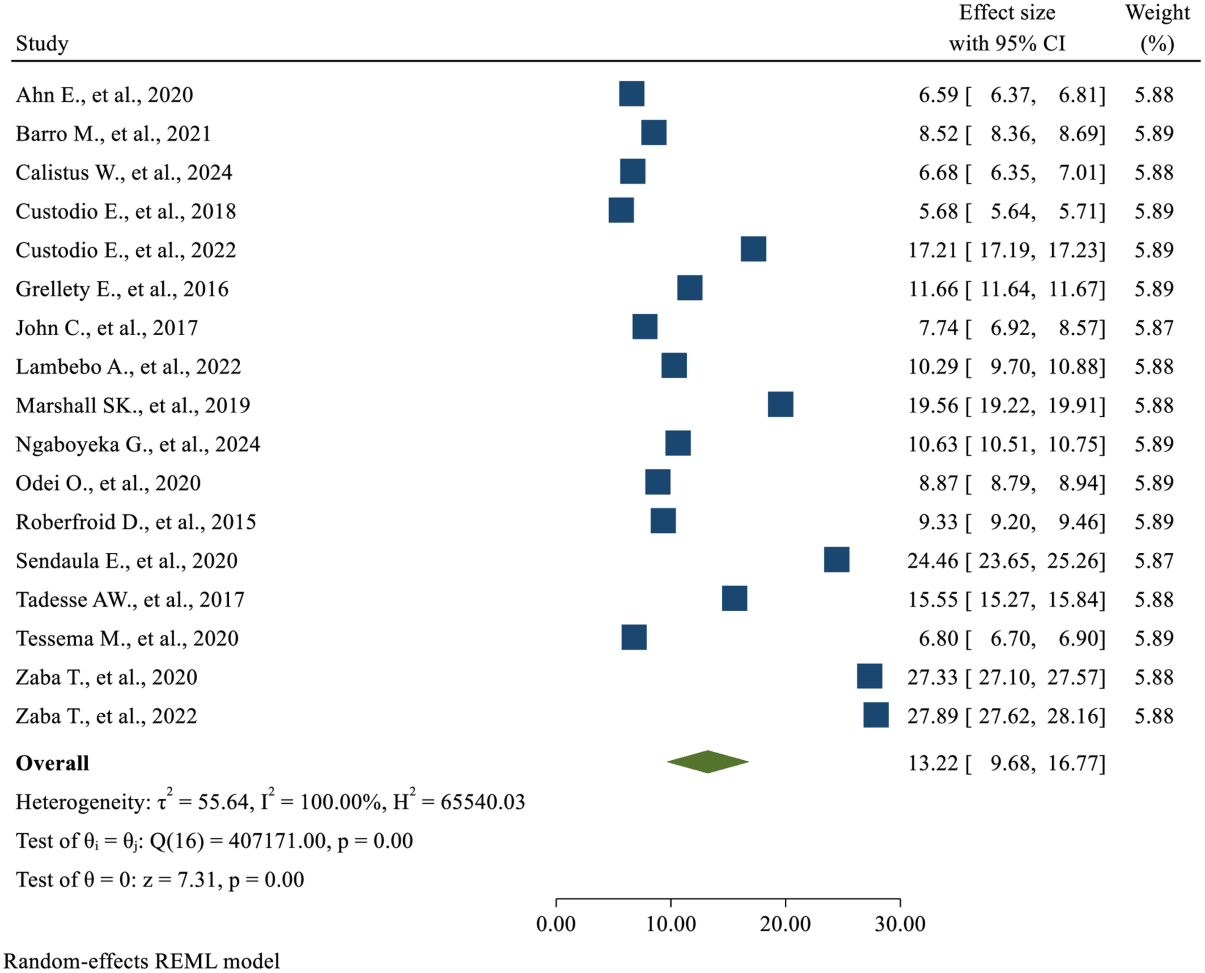
Forest plot—meta-analysis of diagnostic accuracy of MUAC based on DOR.

While analyzing the pooled diagnostic accuracy of MUAC using Diagnostic Odds Ratio (DOR), significantly high heterogeneity was revealed across studies (*I*^2^ = 100%, *p* < 0.01), which means that using a fixed-effects model would have led to an unreliable estimate. Therefore, a random-effect model was a better fit to the data (*I*^2^ = 100%, *p* < 0.01) and the pooled level of DOR was 13.22 (95% CI: 9.68, 16.77) (Figure 7).

### Meta-regression and subgroup analysis

**Figure 8:**
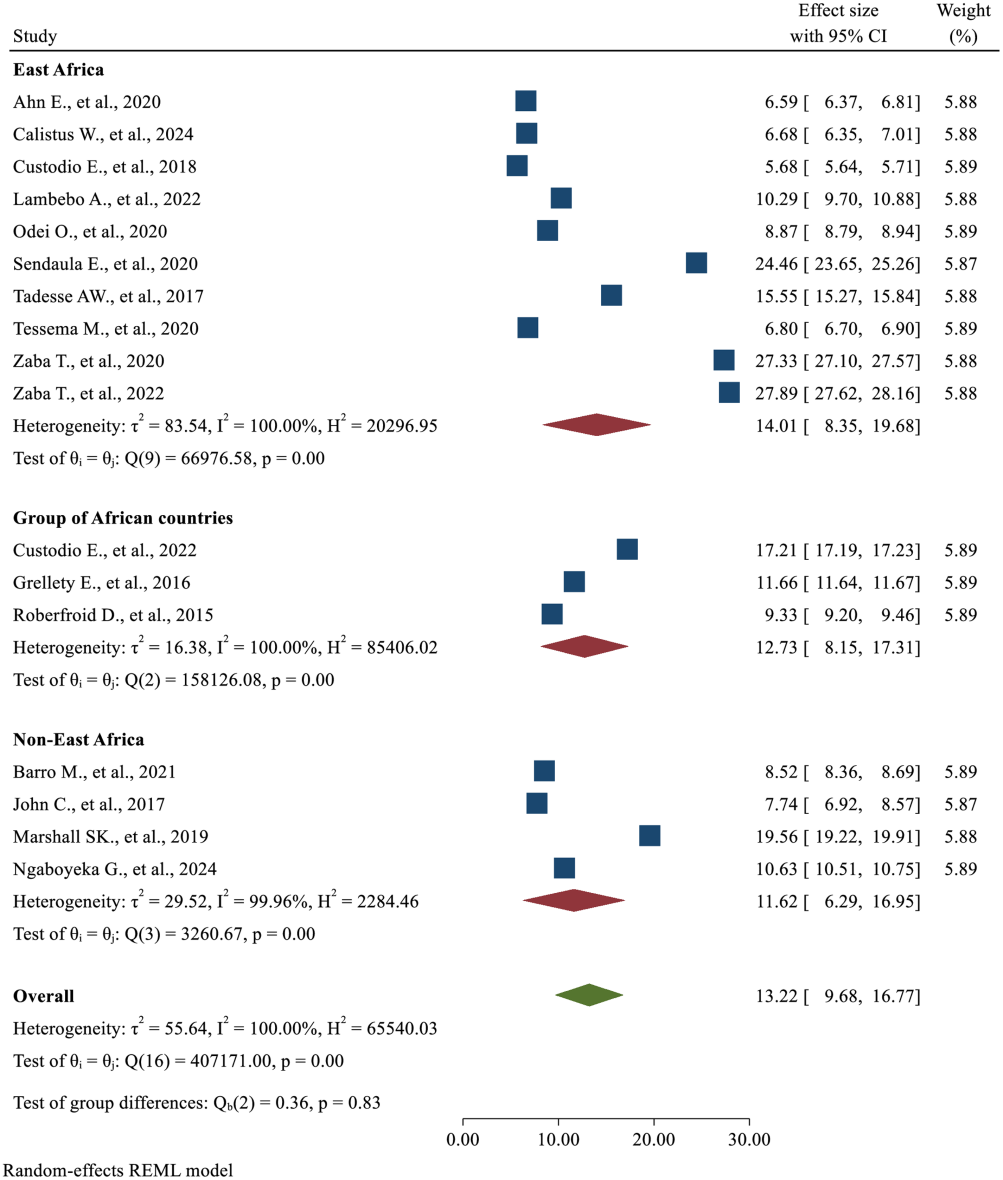
Subgroup analyses by country’s region based on DOR.

**Figure 9:**
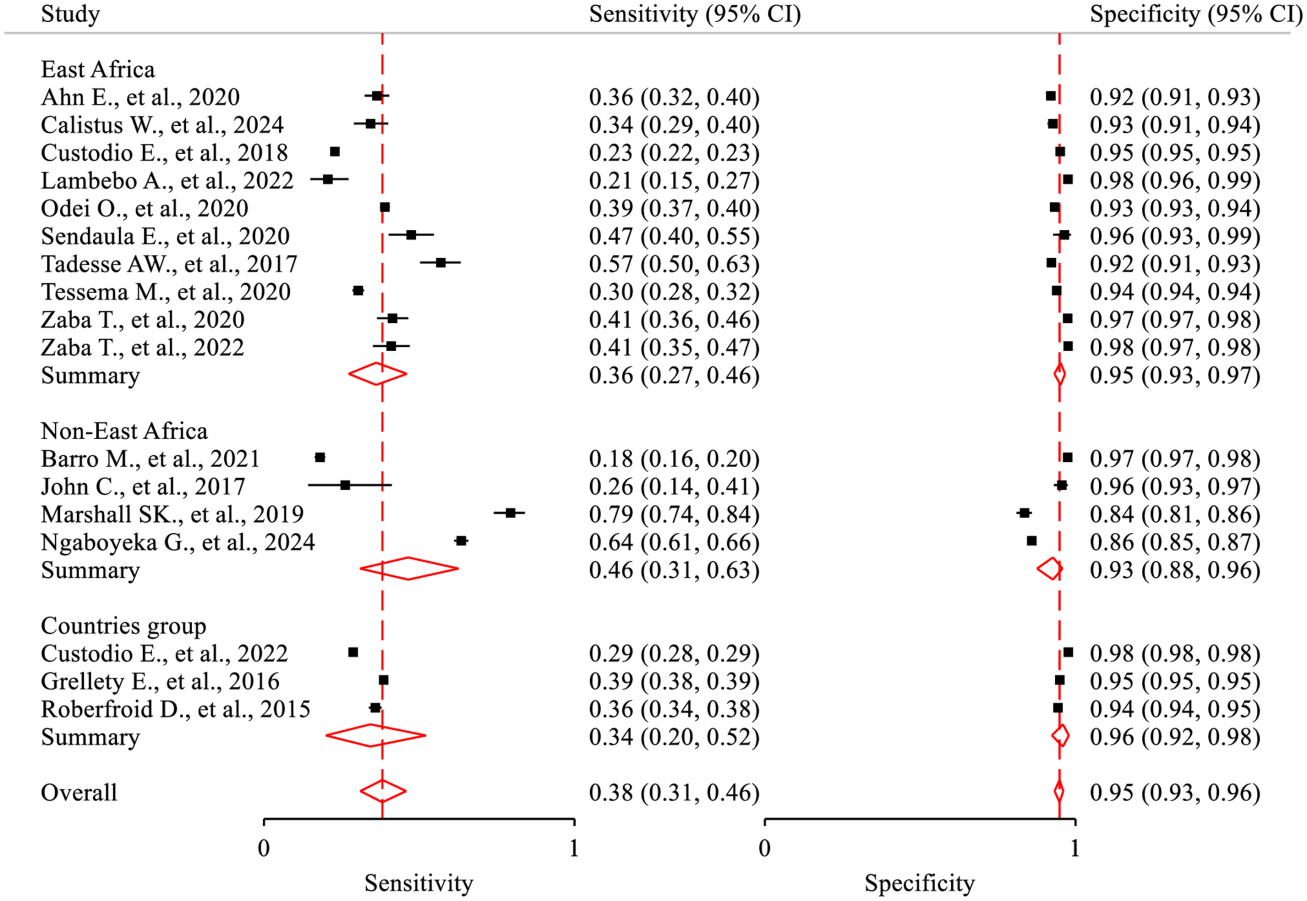
Subgroup analyses by country’s region based on sensitivity and specificity.

A meta-regression analysis was performed to investigate the potential sources of heterogeneity using a univariate regression model, which considered the sample size as a factor. However, no statistically significant findings were observed (*p*-value = 0.890). Subgroup analysis was also conducted using the *metan* package based on selected variables, such as the country’s region, study setting, and sample size. The results of the subgroup analysis indicated a relatively comparable level of DOR across the groups (Table 2; Figure 8). The pooled sensitivity (Sp = 46.4, 95%CI: 31.0, 62.6) in the Non-East Africa Region was relatively higher than in the East Africa Region (Se = 36.1, 95%CI: 27.3, 46.0). On the contrary, the pooled specificity showed a slight decrement while going from the East Africa Region (Sp = 95.2, 95%CI: 93.2, 96.7) to the Non-East Africa Region (Sp = 92.7; 95%CI: 87.6, 95.8) (Table 2; Figure 9).

**Table 2:** Subgroup analyses for included studies.

Subgroups	Number of studies	DOR				Se				Sp			
		DOR (95% CI)	*I*^2^ (%)	*p*-value	Test of group differences	Se (95% CI)	*I*^2^ (%)	*p*-value	Test of group differences	Sp (95% CI)	*I*^2^ (%)	*p*-value	Test of group differences
Sample size													
<10000	9	14.38 (9.18, 19.58)	99.97	<0.01	*Q* = 0.45 *p* = 0.50	45.1 (32.8, 57.4)	99.77	<0.01	*Q* = 3.66 *p* = 0.06	92.7 (89.4, 95.9)	99.16	<0.01	*Q* = 2.67 *p* = 0.10
≥10000	8	11.92 (6.97, 16.88)	100	<0.01		31.8 (26.0, 37.6)	99.98	<0.01		95.5 (94.3, 96.8)	99.94	<0.01	
Setting													
Community-based	14	13.00 (9.08, 16.92)	100	<0.01	*Q* = 0.05 *p* = 0.82	37.3 (29.1, 45.5)	99.99	<0.01	*Q* = 0.52p = 0.47	94.4 (92.5, 96.2)	99.97	<0.01	*Q* = 0.24p = 0.62
Facility based	3	14.27 (4.17, 24.38)	99.89	<0.01		45.7 (24.4, 67.0)	99.17	<0.01		92.6 (86.0, 99.3)	98.3	<0.01	
Country’s region													
East Africa	10	14.01 (8.35, 19.68)	100	<0.01	*Q* = 0.36 *p* = 0.83	36.1 (27.3, 46.0)	99.89	<0.01	*Q* = 0.91 *p* = 0.64	95.2 (93.2, 96.7)	99.49	<0.01	*Q* = 2.01 *p* = 0.37
Group of African countries	3	12.73 (8.15, 17.31)	100	<0.01		34.2 (19.9, 52.1)	99.98	<0.01		96.0 (92.4, 97.9)	99.98	<0.01	
Non-East Africa	4	11.62 (6.29, 16.95)	99.96	<0.01		46.4 (31.0, 62.6)	99.94	<0.01		92.7 (87.6, 95.8)	99.54	<0.01	
Overall	17	13.22 (9.68, 16.77)	100	<0.01		38.1 (30.7, 46.1)	99.99	<0.01		94.9 (93.2, 96.2)	99.97	<0.01	

DOR, diagnostic odds ratio; Se, sensitivity; Sp, specificity.

### Sensitivity analysis

**Figure 10:**
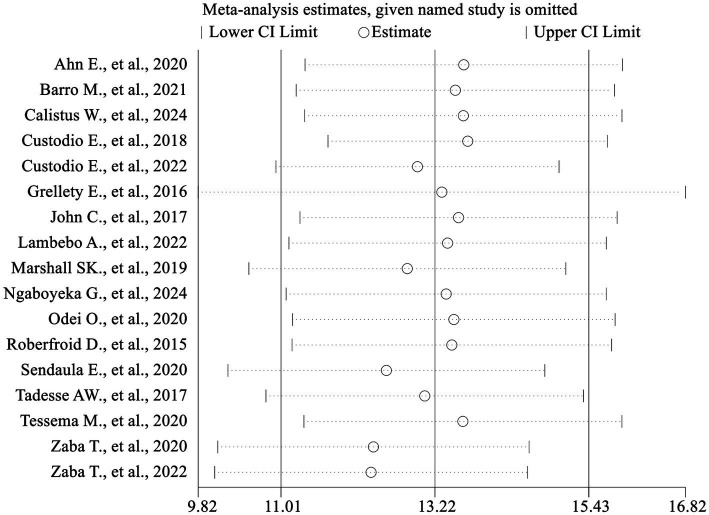
Sensitivity analysis.

A sensitivity analysis was conducted to demonstrate the influence of individual studies on the overall estimates of the meta-analysis. The point estimate of an individual study “omitted” analysis lies inside the confidence interval of the “combined” analysis. This analysis indicated that the meta-analysis summary estimate was robust and not dependent on any one study (Table 3; Figure 10).

**Table 3:** Sensitivity analysis based on DOR.

Study omitted	Estimate (DOR)	95% CI
Ahn (22)	13.64	11.36–15.92
Barro (23)	13.51	11.23–15.80
Calistus (13)	13.63	11.35–15.91
Custodio et al. (24)	13.69	11.69–15.70
Custodio et al. (1)	12.97	10.94–15.00
Grellety et al.(2)	13.32	9.82–16.82
John et al. (25)	13.56	11.28–15.84
Lambebo et al. (19)	13.40	11.12–15.68
Marshall et al.(26)	12.82	10.55–15.10
Ngaboyeka et al. (27)	13.38	11.08–15.68
Odei et al. 2020 (28)	13.49	11.18–15.81
Roberfroid et al. (7)	13.46	11.17–15.76
Sendaula et al. (11)	12.52	10.25–14.80
Tadesse et al. (20)	13.07	10.79–15.36
Tessema et al. (21)	13.62	11.34–15.91
Zaba et al. 2020 (29)	12.34	10.10–14.58
Zaba et al. (30)	12.30	10.06–14.55

DOR, diagnostic odds ratio; CI, confidence interval.

### Publication bias

**Figure 11:**
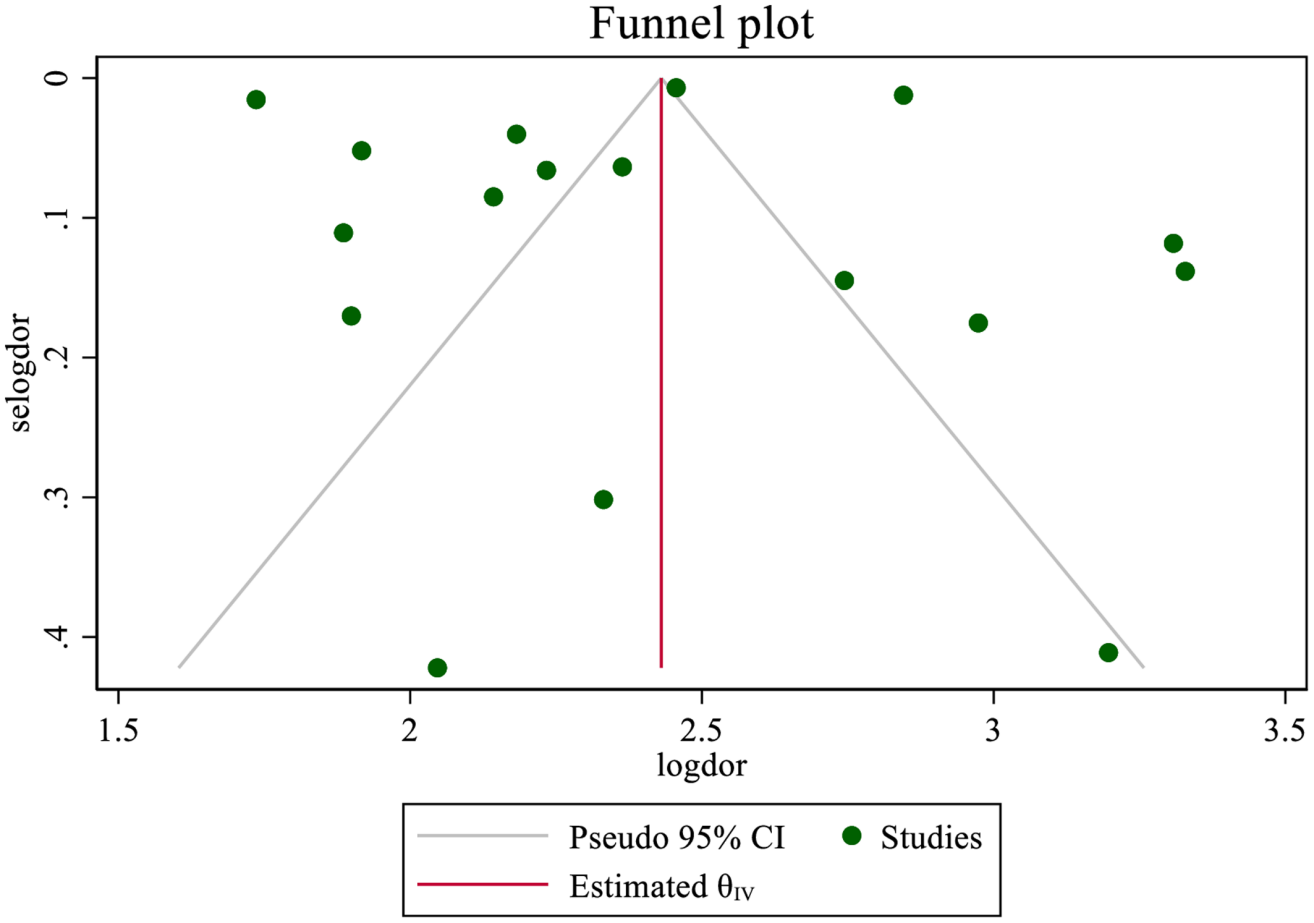
Funnel plot—meta-analysis of diagnostic accuracy of MUAC to diagnose acute malnutrition using DOR among children aged 6–59 months in Africa.

The publication bias was checked by Egger’s test and funnel plot of asymmetry using the DOR as effect size. Funnel plots designed separately for sensitivity and specificity (following a logit transformation) are probably not very effective in identifying sample size effects. Interpreting the two associated funnel plots along with the two tests for assessing asymmetry can be quite challenging. Therefore, the analysis was limited to funnel plots derived from the DOR. The result of Egger’s test (*p* = 0.391) suggested that there was no publication bias. There was a symmetric pattern around the overall effect, which indicated no publication bias, even though most of the studies fell outside the 95% CI (Figure 11).

## Discussion

This systematic review and meta-analysis were conducted to assess the diagnostic test accuracy of MUAC against WHZ in screening acute malnutrition among children aged 6–59 months in Africa. This review indicated that the pooled sensitivity and specificity of MUAC in detecting acute malnutrition were found to be 38.1 and 94.9%, respectively. The limited sensitivity and high specificity of MUAC have been reported in another systematic review and meta-analysis (8) and original research in India (31), Nepal (32) and Cambodia (33). However, another study in India revealed that MUAC showed greater sensitivity (63.7%) while maintaining comparable specificity (95.8%) (34). Similarly, a study conducted in Bangladesh reported a higher sensitivity of 63.2%, although the specificity was comparatively lower at 85.3% (35). The observed disparity may stem from a complex interplay of biological, cultural, and environmental factors that differ across racial and ethnic groups, which can result in variations in anthropometric dimensions associated with the body frame.

This low sensitivity has significant implications for screening initiatives and nutritional management. Inadequate identification and missing of undernourished children can result in delayed diagnoses, exacerbating severe health complications and even leading to fatalities. Failure to recognize malnourished individuals may have enduring consequences on their health and overall quality of life. Additionally, inaccurate diagnoses in children could result in inefficient allocation of resources and ineffective interventions. One possible explanation for this low sensitivity may lie in the MUAC cutoff threshold; increasing this cutoff may potentially enhance sensitivity. The standard MUAC cutoffs may not adequately capture the full spectrum of malnutrition across different populations. Increasing the MUAC cutoff threshold could potentially enhance sensitivity by identifying a broader range of at-risk children who might otherwise be overlooked. However, raising the cutoff also poses the risk of reducing specificity, which could lead to an increase in false positives and place additional strain on already limited resources. Therefore, adjusting cutoffs should be carefully evaluated and validated within specific contexts to balance sensitivity and specificity, ensuring the accurate identification of malnourished children and enhancing the effectiveness of interventions (6).

The MUAC showed an 11.7% misclassification rate when screening for acute malnutrition. This designates that a considerable number of children were improperly classified, leading to many being overlooked in the screening process and incorrectly deemed free of acute malnutrition, while some others were erroneously diagnosed as malnourished. Such a misclassification of nearly one in ten children poses a significant public health concern.

This study revealed that MUAC was a more precise measurement compared to its sensitivity. Children diagnosed with acute malnutrition based on MUAC have a significant likelihood of also being affected by acute malnutrition, as validated by WHZ. This indicates that employing MUAC as a screening tool was efficient for treatment, ultimately saving time and resources by lowering the incidence of false positives in acute malnutrition assessments.

The diagnostic test accuracy of MUAC for acute malnutrition was good (AUC = 0.85). This discriminatory performance was comparable to findings from other original research conducted in Asia (31, 32, 34, 36) and higher than study in Cambodia (33). The strong accuracy of this simple and user-friendly measurement was encouraging and suggested that MUAC could potentially represent WHZ assessments in both community and clinical settings. Furthermore, this level of performance could be further improved to achieve excellent diagnostic test accuracy with appropriate adjustments to the cutoff values.

The pooled DOR of 13.22 indicates that MUAC demonstrates high discriminatory performance. A higher DOR signifies that MUAC is more effective at distinguishing between individuals with and without acute malnutrition, the condition of interest. As a measure of test performance, the DOR combines sensitivity and specificity, providing prevalence-independent indicators while also reflecting overall accuracy in a single metric. This means that MUAC measurements are well-suited for identifying children with acute malnutrition or those in need of intervention.

Although not statistically significant, the visible pooled sensitivity in the Non-East Africa Region (46.4%) was marginally higher than in the East Africa Region (36.1%), while the pooled specificity slightly decreased from 95.2% in the East Africa Region to 92.7% in the Non-East Africa Region. These differences may be explained by variations in body composition and anthropometric characteristics between regions, which can influence the accuracy of MUAC as a screening tool. Differences in body frame, such as muscle mass, fat distribution, and overall arm circumference, could lead to variations in how malnutrition is detected and classified across diverse populations.

This meta-analysis faced a limitation due to notable heterogeneity between the studies. To address this, a meta-regression and subgroup analysis were performed, taking potential variables into account. A key strength of this study lies in its use of consistent cutoff values for both the index test and the reference standard, effectively eliminating any bias related to diagnostic thresholds. Furthermore, potential performance indicators were executed to pool the diagnostic accuracy of MUAC assessments across various studies conducted in multiple African countries.

### Conclusion

The MUAC demonstrated low sensitivity but high specificity in diagnosing acute malnutrition in children aged 6 to 59 months across various regions of Africa. Furthermore, it was found that MUAC provides good diagnostic test accuracy when compared to WHZ. To enhance its accuracy, it is suggested to increase the MUAC cutoff thresholds.
